# Approximating Ideal Filters by Systems of Fractional Order

**DOI:** 10.1155/2012/365054

**Published:** 2012-01-16

**Authors:** Ming Li

**Affiliations:** ^1^School of Information Science & Technology, East China Normal University, No. 500, Dong-Chuan Road, Shanghai 200241, China; ^2^Department of Computer and Information Science, University of Macau, Avenue Padre Tomas Pereira, Taipa 1356, Macau, China

## Abstract

The contributions in this paper are in two folds. On the one hand, we propose a general approach for approximating ideal filters based on fractional calculus from the point of view of systems of fractional order. On the other hand, we suggest that the Paley and Wiener criterion might not be a necessary condition for designing physically realizable ideal filters. As an application of the present approach, we show a case in designing ideal filters for suppressing 50-Hz interference in electrocardiogram (ECG) signals.

## 1. Introduction

Filters have wide applications in various fields, ranging from medical engineering to electrical engineering; see, for example, Hussain et al. [1], Bhattacharyya et al. [2], Fieguth [3], Bendat and Piersol [4], Gray and Davisson [5], and Li [6], just mentioning a few. In the field, the theory and techniques to approximate ideal filters are desired. There are some methods about approximating ideal filters, such as Butterworth filters, Chebyshev filters, Cauer-Chebyshev filters, and Bessel ones (Wanhammar [7], Mitra and Kaiser [8]).

Recall that the conventional filters of Butterworth type, Chebyshev type, Cauer-Chebyshev type, or Bessel one are discussed in the domain of systems of integer order. More precisely, the frequency response of a filter that is denoted by *H*(*ω*) is a rational function. Both the denominator and the numerator of the rational function are polynomials of integer order; see [7, 8], Vegte [9], Dorf and Bishop [10], and Li [11]. From the point of view of mathematical analysis, conventional filters are in the domain of calculus of integer order.

This paper aims at providing an approach to approximate ideal filters by using frequency responses of fractional-order. The basic idea is like this. Denote by *ω*_*c*_ the cutoff frequency of a filter. Then, *H*(*ω*_*c*_) = 0 from a view of ideal filters. In this case, we present the following approximation:

(1) $$\underset{r\to 0}{\lim}{\left|H\left(\omega \right)\right|}^{r}=\{\begin{array}{cc} 1, & \omega \neq \omega_{c},\\ 0, & \omega =\omega_{c}, \end{array}$$

where |*H*(*ω*)| is the amplitude of *H*(*ω*).

An obvious advantage of the present approach is that the above always holds no matter what the concrete structure of *H*(*ω*) is. However, theoretically speaking, *H* ^*r*^(*ω*) has to be explained from the point of view of fractional calculus.

The remaining paper is organized as follows.  Section 2 explains the research background. The problem statement is described in Section 3. The present approximation is given in Section 4. A case study is stated in Section 5, which is followed by conclusions.

## 2. Research Background

### 2.1. Glimpse at Ideal Filters

The ideal lowpass filter implies that the amplitude of the frequency response is given by

(2) $$\left|H\left(\omega \right)\right|=\{\begin{array}{cc} 1, & \omega <\omega_{c}\\ 0, & \text{elsewhere,} \end{array}$$

where *H*(*ω*_*c*_) = 0. One says that *H*(*ω*) is the frequency response of an ideal highpass filter if

(3) $$\left|H\left(\omega \right)\right|=\{\begin{array}{cc} 1, & \omega >\omega_{c},\\ 0, & \text{elsewhere.} \end{array}$$

The ideal bandpass filter has the frequency response expressed by

(4) $$\left|H\left(\omega \right)\right|=\{\begin{array}{cc} 1, & \omega_{{\text{c}}_{\text{l}}}<\omega <\omega_{{\text{c}}_{\text{h}}},\\ 0, & \text{elsewhere,} \end{array}$$

where *ω*_c_l__ and *ω*_c_h__ are cut-off frequencies. A filter is said to be ideal band stop if its frequency response function is given by

(5) $$\left|H\left(\omega \right)\right|=\{\begin{array}{cc} 0, & \omega_{{\text{c}}_{\text{l}}}<\omega <\omega_{{\text{c}}_{\text{h}}},\\ 1, & \text{elsewhere.} \end{array}$$

### 2.2. Paley and Wiener Criterion

For facilitating the discussions, we write

(6) $$\begin{array}{c} H\left(\omega \right)=R\left(\omega \right)+jX\left(\omega \right)=A\left(\omega \right)e^{-j\vartheta (\omega )}, \end{array}$$

where *ϑ*(*ω*) is the phase response of a filter. Note that the condition for *F* ^−1^[*H*(*ω*)] = *h*(*t*) to be zero for negative *t*, where *F* ^−1^ implies the inverse of the Fourier transform, is that *A*(*ω*) must be square-integrable. That is,

(7) $$\begin{array}{c} \overset{\infty }{\underset{-\infty }{\int }}A^{2}\left(\omega \right)d\omega <\infty . \end{array}$$

The above implies the causality of a filter; see, for example, Papoulis [12]. A necessary and sufficient condition for *A*(*ω*) to satisfy (7) is explained by Paley and Wiener [13]. That condition is called the Paley and Wiener condition or the Paley and Wiener criterion. It is expressed by

(8) $$\begin{array}{c} \overset{\infty }{\underset{-\infty }{\int }}\frac{\left|\ln A\left(\omega \right)\right|}{1+\omega^{2}}d\omega <\infty . \end{array}$$

The Paley and Wiener criterion implies that ideal filters are not physically realizable because *A*(*ω*) = 0 in a certain frequency range for each type of ideal filters. Therefore, approximations of ideal filters are desired.

### 2.3. Some Filters of Integer Order for Approximating Ideal Filters

Various methods in the approximations are studied, such as Butterworth filters, Chebyshev's, Cauer-Chebyshev's, and Bessel's filters; see, for example, [2], and Lam [14].

Taking lowpass filtering as an example, the system function of the Butterworth filters of order *n* is given by

(9) $$\begin{array}{c} A\left(\omega \right)=\frac{1}{\sqrt{1+{\left(\omega /\omega_{c}\right)}^{2n}}},\;n=1,2,\ldots . \end{array}$$

Denote the Chebyshev polynomial of the first kind by *C*_*n*_(*ω*). Then,

(10) $$\begin{array}{c} C_{n}\left(\omega \right)=\{\begin{array}{cc} \begin{array}{cc} \cos\left(n\;\cos^{-1}\frac{\omega }{\omega_{c}}\right), & \left|\omega \right|\leq \omega_{c},\\ \text{ch}\left(n\;{\text{ch}}^{-1}\frac{\omega }{\omega_{c}}\right), & \left|\omega \right|>\omega_{c}, \end{array} & n=1,2,\ldots . \end{array} \end{array}$$

The frequency response of the Chebyshev type lowpass filters for *ε* > 0 is given by

(11) $$\begin{array}{c} A\left(\omega \right)=\frac{1}{\sqrt{1+\varepsilon^{2}C_{n}^{2}\left(\omega \right)}},\;n=1,2,\ldots . \end{array}$$

Denote the Chebyshev rational function of degree *n* by *R*_*n*_(*ω*). Then,

(12) $$\begin{array}{c} R_{n}\left(\omega \right)=C_{n}\left(\frac{\omega -1}{\omega +1}\right),\;n=1,2,\ldots . \end{array}$$

One of the applications of *R*_*n*_(*ω*) is to design an elliptic filter, which is also known as a Cauer filter, named after Wilhelm Cauer. An elliptic filter has the property of equalized ripple (equiripple) behavior in both the passband and the stopband. The frequency response of the elliptic type lowpass filters for *ε* > 0 is given by

(13) $$\begin{array}{c} A\left(\omega \right)=\frac{1}{\sqrt{1+\varepsilon^{2}R_{n}^{2}\left(\xi ,\omega /\omega_{c}\right)\;}},\;n=1,2,\ldots , \end{array}$$

where *ε* is the ripple factor, and *ξ* is the selectivity factor [15, 16].

## 3. Problem Statement

The Butterworth filters obviously correspond to linear differential equations of integer order [17, 18].

Note that the Chebyshev polynomial of the first kind is the solution to the Chebyshev equation that is the second-order linear differential equation given by

(14) $$\begin{array}{c} \left(1-x^{2}\right)\frac{d^{2}y}{dx^{2}}-x\frac{dy}{dx}+n^{2}y=0. \end{array}$$

Therefore, a consequence we note hereby is that the Chebyshev type filters as well as the elliptic type filters are corresponding to linear differential equations of integer order.

Recently, filters of fractional-order attract much attention in the field of circuits, systems, and signals; see, for example, Podlubny [19], Ortigueira [20], MacHado et al. [21], Lim et al. [22], Chen and Moore [23], and Zhang [24], simply citing a few. However, the literature regarding approximating ideal filters from a view of filters of fractional-order is rarely seen. For that reason, we propose a question like this. May ideal filters be approximated by filters or equations of fractional-order? We will give the affirmative answer to it in the next section.

## 4. Approximating Ideal Filters by Systems of Fractional Order

A linear filter can be expressed by a linear differential equation given by

(15) $$\begin{array}{c} \overset{p}{\underset{i=0}{\sum }}a_{i}\frac{d^{p-i}y\left(t\right)}{dt^{p-i}}=\overset{q}{\underset{i=0}{\sum }}b_{i}\frac{d^{q-i}e\left(t\right)}{dt^{q-i}}, \end{array}$$

where *y*(*t*) is the response and *e*(*t*) excitation. Denote the Fourier transforms of *y*(*t*) and *e*(*t*) by *Y*(*ω*) and *E*(*ω*), respectively. Then, the system function is given by

(16) $$\begin{array}{c} H\left(\omega \right)=\frac{Y\left(\omega \right)}{E\left(\omega \right)}. \end{array}$$

Denote |*H*(*ω*)| by *A*(*ω*). Then, *H*(*ω*) = *A*(*ω*)*e* ^−*jϑ*(*ω*)^. This is the basic principle regarding linear filters. In this case, we say that *H*(*ω*) is the system function or frequency response of a filter of integer order; see, for example, Monje et al. [25].

We now consider a filter of fractional-order presented by

(17) $$\begin{array}{c} H_{1}\left(\omega \right)={\left[H\left(\omega \right)\right]}^{r}={\left[A\left(\omega \right)\right]}^{r}e^{-jr\vartheta (\omega )}\;\left(r>0\right). \end{array}$$

Denote

(18) $$\begin{array}{c} H_{1}\left(\omega \right)=A_{1}\left(\omega \right)e^{-j\vartheta_{1}(\omega )}. \end{array}$$

Then,

(19) $$\begin{array}{c} A_{1}\left(\omega \right)={\left[A\left(\omega \right)\right]}^{r},\;\;\vartheta_{1}\left(\omega \right)=r\vartheta \left(\omega \right). \end{array}$$

Since *ϑ*_1_(*ω*) is similar to *ϑ*(*ω*), the key difference between *H*(*ω*) and *H*_1_(*ω*) is in the aspect of amplitude response, namely, *A*(*ω*) and *A*_1_(*ω*).

It can be seen from (19) that

(20) $$\begin{array}{c} \underset{r\to 0}{\lim}A_{1}\left(\omega \right)=\underset{r\to 0}{\lim}{\left[A\left(\omega \right)\right]}^{r}=1\;\text{for}\;A\left(\omega \right)\neq 0. \end{array}$$

In addition,

(21) $$\begin{array}{c} \underset{r\to 0}{\lim}A_{1}\left(\omega_{c}\right)=\underset{r\to 0}{\lim}{\left[A\left(\omega_{c}\right)\right]}^{r}=0\;\text{if}\;A\left(\omega_{c}\right)=0. \end{array}$$

Denote *B*_0.7_ the 3-dB bandwidth of *H*_1_(*ω*) by

(22) $$\begin{array}{c} {A_{1}\left(f\right)|}_{f=B_{0.7}}=0.707, \end{array}$$

where *f* = *ω*/2*π*  is frequency. Denote *B*_0.1_ the bandwidth for

(23) $$\begin{array}{c} {A_{1}\left(f\right)|}_{f=B_{0.1}}=0.1. \end{array}$$

Then, the rectangular coefficient defined by

(24) $$\begin{array}{c} \text{Rec}=\frac{B_{0.7}}{B_{0.1}} \end{array}$$

is always ideal for *A*_1_(*ω*). That is,

(25) $$\begin{array}{c} \text{Rec}=\frac{B_{0.7}}{B_{0.1}}=1\;\text{for}\;A_{1}\left(\omega \right), \end{array}$$

because of (20).

On the other hand,

(26) $$\begin{array}{c} \underset{r\to 0}{\lim}\vartheta_{1}\left(\omega \right)=0. \end{array}$$

The expression (26) implies that *H*_1_(*ω*) always has a linear phase response.

**Remark 1**

Equation (25) does not relate to any concrete forms of *H*_1_(*ω*). Thus, the present results, namely, (20) and (21), stand for a general approach for approximating ideal filters based on systems of fractional-order.

**Remark 2**

Let

(27) $$\begin{array}{c} H_{0}\left(\omega \right)=\underset{r\to 0}{\lim}{\left[H\left(\omega \right)\right]}^{r},\;\;A_{0}\left(\omega \right)=\underset{r\to 0}{\lim}{\left[A\left(\omega \right)\right]}^{r}. \end{array}$$

Then, *A*_0_(*ω*) does not satisfy the Paley and Wiener criterion expressed by (8) because

(28) $$\begin{array}{c} A_{0}\left(\omega \right)=\{\begin{array}{cc} 1 & \text{if}\;A_{0}\left(\omega \right)\neq 0,\\ 0 & \text{if}\;A_{0}\left(\omega \right)=0. \end{array} \end{array}$$

That is,

(29) $$\begin{array}{c} \overset{\infty }{\underset{-\infty }{\int }}\frac{\left|\ln A_{0}\left(\omega \right)\right|}{1+\omega^{2}}d\omega =\infty . \end{array}$$

Therefore, this remark suggests a theoretical significance that the Paley and Wiener criterion might not be a necessary condition for designing physically realizable ideal filters of fractional-order.

## 5. Case Study

**Figure 1:**
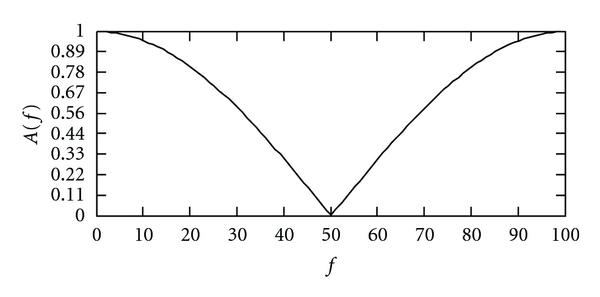
Amplitude response of filter.

We consider a finite impulse response filter (FIR) given by

(30) $$\begin{array}{cc} H\left(f\right) & =\frac{1+\cos\left(2\pi fT\right)-j\sin\left(2\pi fT\right)}{2}\\ & =\frac{1}{2}\left[1+\exp\left(-j2\pi fT\right)\right],\;j=\sqrt{-1}, \end{array}$$

where *T* is the sampling period.  Figure 1 indicates *A*(*f*) for *T* = 0.01.

For *A*(*f*) = |*H*(*f*)| and *T* = 0.01, we have

(31) $${A\left(f\right)|}_{f=50}=0.$$

Note that

(32) $$\begin{array}{c} {A\left(f\right)|}_{f=25}=0.707,\\ {A\left(f\right)|}_{f=46.8}=0.1, \end{array}$$

Thus, the rectangular coefficient of *H*(*f*) is

(33) $$\frac{B_{0.7}}{B_{0.1}}=\frac{25}{46.8}=0.534.$$

The rectangular coefficient of 0.534 exhibits that *H*(*f*) is not a satisfactory filter in general. Nevertheless, one is able to easily modify it to be such that it is an ideal filter by

(34) $$\begin{array}{c} \underset{r\to 0}{\lim}{\left[H\left(f\right)\right]}^{r}=\underset{r\to 0}{\lim}{\left[\frac{1+\exp\left(-j2\pi fT\right)}{2}\right]}^{r}=\{\begin{array}{cc} 1, & f<50,\\ 0, & f=50. \end{array} \end{array}$$

**Figure 2:**
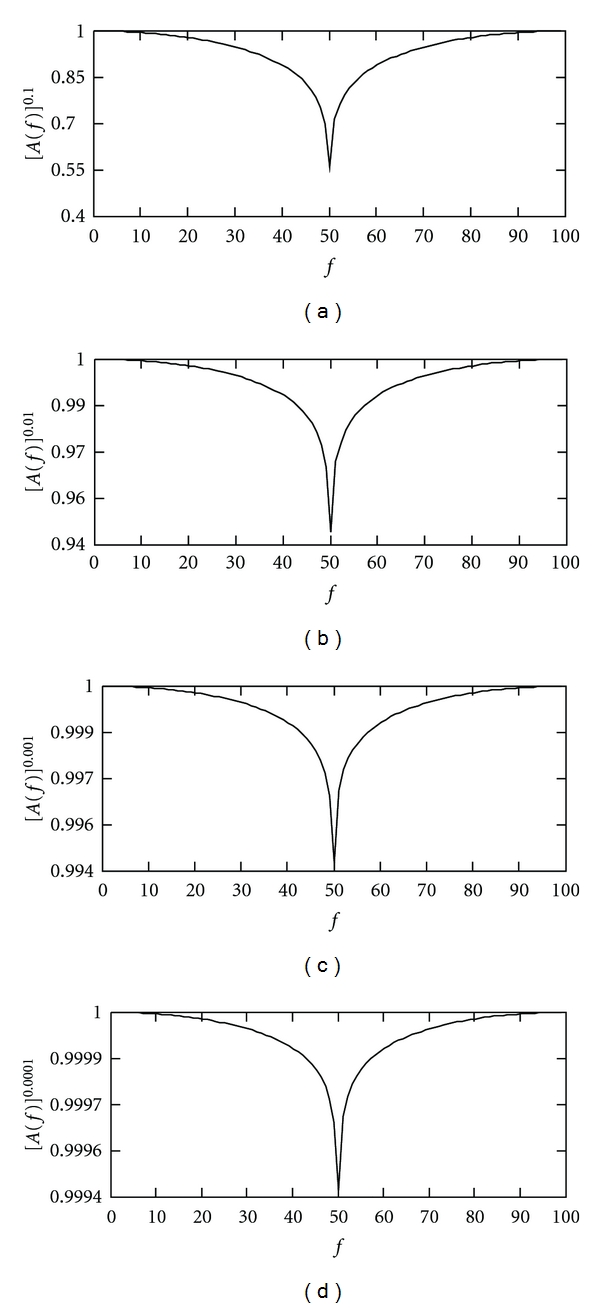
Approximations of [*H*(*f*)]^*r*^ for different values of *r*. (a) *r* = 0.1. (b) *r* = 0.01. (c) *r* = 0.001. (d) *r* = 0.0001.

Figure 2 shows the approximations of [*H*(*f*)]^*r*^ for *r* = 0.1, 0.01, 0.001, and 0.0001, respectively. It exhibits that the present method well approximates the ideal filter. As a matter of fact, in the sense of 0.9994 *≈* 1 [*H*(*f*)]^*r*^ for *r* = 0.0001, see Figure 2(d), can be regarded as an ideal filter in practice.

The following is called a binomial series:

(35) $$\begin{array}{c} {\left(1+x\right)}^{\nu }=\overset{\infty }{\underset{k=0}{\sum }}\left(\begin{array}{c} \nu \\ k \end{array}\right)\;x^{k}\;\text{for}\;\left|x\right|<1, \end{array}$$

where $\left(\begin{array}{c} \nu \\ k \end{array}\right)=\Gamma (\nu +k)/\Gamma (\nu )\Gamma (1+k)$ is binomial coefficient [26]. By using binomial series, (34) can be expanded by

(36) $$\begin{array}{cc} \underset{r\to 0}{\lim}{\left[H\left(f\right)\right]}^{r} & =\underset{r\to 0}{\lim}{\left[\frac{1+\exp\left(-j2\pi fT\right)}{2}\right]}^{r}\\ & =\frac{1}{2}\underset{r\to 0}{\lim}\overset{\infty }{\underset{k=0}{\sum }}\frac{\Gamma \left(r+k\right)}{\Gamma \left(r\right)\Gamma \left(1+k\right)}\exp\left(-jk2\pi fT\right). \end{array}$$

Therefore, in general, [*H*(*f*)]^*r*^ should be taken as a filter of fractional-order from a view of fractional-order systems [25]; see the Appendix for the meaning of [*H*(*f*)]^*r*^ in fractional-order systems.

It is worth noting that (34) may yet be an ideal FIR notch filter used for suppressing 50-Hz interference in electrocardiogram (ECG) signals, which is a key component in processing ECG signals in medical engineering; see, for example, Talmaon [27], Levkov et al. [28], Martens et al. [29], Dotsinsky and Stoyanov [30], and Li [31], though *H*(*f*) is not a satisfactory filter for this purpose. Finally, it is noted that the research though reflected in this paper might be used for studying other topics, such as those in [32–35].

## 6. Conclusions

We have presented a general approach for approximating ideal filters from a view of fractional-order systems. This approach is based on fractional calculus. The theoretical significance of the present approach is that the Paley and Wiener criterion might be no longer a necessary condition for designing physically realizable ideal filters. We have showed a case that can be used for designing ideal filters for suppressing 50-Hz interference in ECG signals.

## Appendix

The fractional derivative of Caputo type of a function *f*(*t*) is defined by

(A.1) $$\begin{array}{c} D_{t}^{\nu }f\left(t\right)=\frac{1}{\Gamma \left(n-\nu \right)}\overset{t}{\underset{0}{\int }}\frac{f^{\left(n\right)}\left(u\right)du}{{\left(t-u\right)}^{\nu -n+1}},\;n-1\leq \alpha \leq n, \end{array}$$

where Γ is the Gamma function [19]. For simplicity, we write _0_^^ *D*_*t*_^*v*^ by *D* ^*v*^. Without generality losing, we take a system of second-order as a case:

(A.2) $$\begin{array}{c} \left(\frac{d^{2}}{dt^{2}}+\omega_{0}^{2}\right)x\left(t\right)=e\left(t\right),\;\omega_{0}>0. \end{array}$$

There are two types of fractional-order systems based on (A.2). One is given by (see [36])

(A.3) $$\begin{array}{c} \left(\frac{d^{2-\varepsilon }}{dt^{2-\varepsilon }}+\omega_{0}^{2}\right)x\left(t\right)=e\left(t\right),\;0<\varepsilon <1. \end{array}$$

Another is expressed by

(A.4) $$\begin{array}{c} {\left(\frac{d^{2}}{dt^{2}}+\omega_{0}^{2}\right)}^{\beta }x\left(t\right)=e\left(t\right),\;\beta >0; \end{array}$$

see, for example, [37, 38].

Denote the impulse response function of (A.5) by *h*(*t*). Then,

(A.5) $$\begin{array}{c} {\left(D^{2}+\omega_{0}^{2}\right)}^{\beta }h\left(t\right)=\delta \left(t\right), \end{array}$$

where *δ*(*t*) is the Dirac-*δ* function.

Denote the Fourier transform of *h*(*t*) by *H*(*ω*). Then, we have

(A.6) $$\begin{array}{c} H\left(\omega \right)=\frac{1}{{\left(\omega^{2}+\omega_{0}^{2}\right)}^{\beta }}. \end{array}$$

Therefore, if one denotes the frequency response of (A.2) by *H*_0_(*ω*), which is a system of 2-order,

(A.7) $$\begin{array}{c} H_{0}\left(\omega \right)=\frac{1}{\left(\omega^{2}+\omega_{0}^{2}\right)}, \end{array}$$

then,

(A.8) $$\begin{array}{c} H\left(\omega \right)={\left[H_{0}\left(\omega \right)\right]}^{\beta }. \end{array}$$

The expression (A.8) gives the explanation of [*H*(*f*)]^*r*^ in fractional-order systems discussed in the body text of the paper.
